# Supplementary figures and images for: Safety and cognitive pharmacodynamics following dose escalations with 3-methylmethcathinone (3-MMC): a first in human, designer drug study
Source: Neuropsychopharmacology. 2024 Dec 24;50(7):1084–92. doi: 10.1038/s41386-024-02042-7 (PMC12089282; doi:10.1038/s41386-024-02042-7)

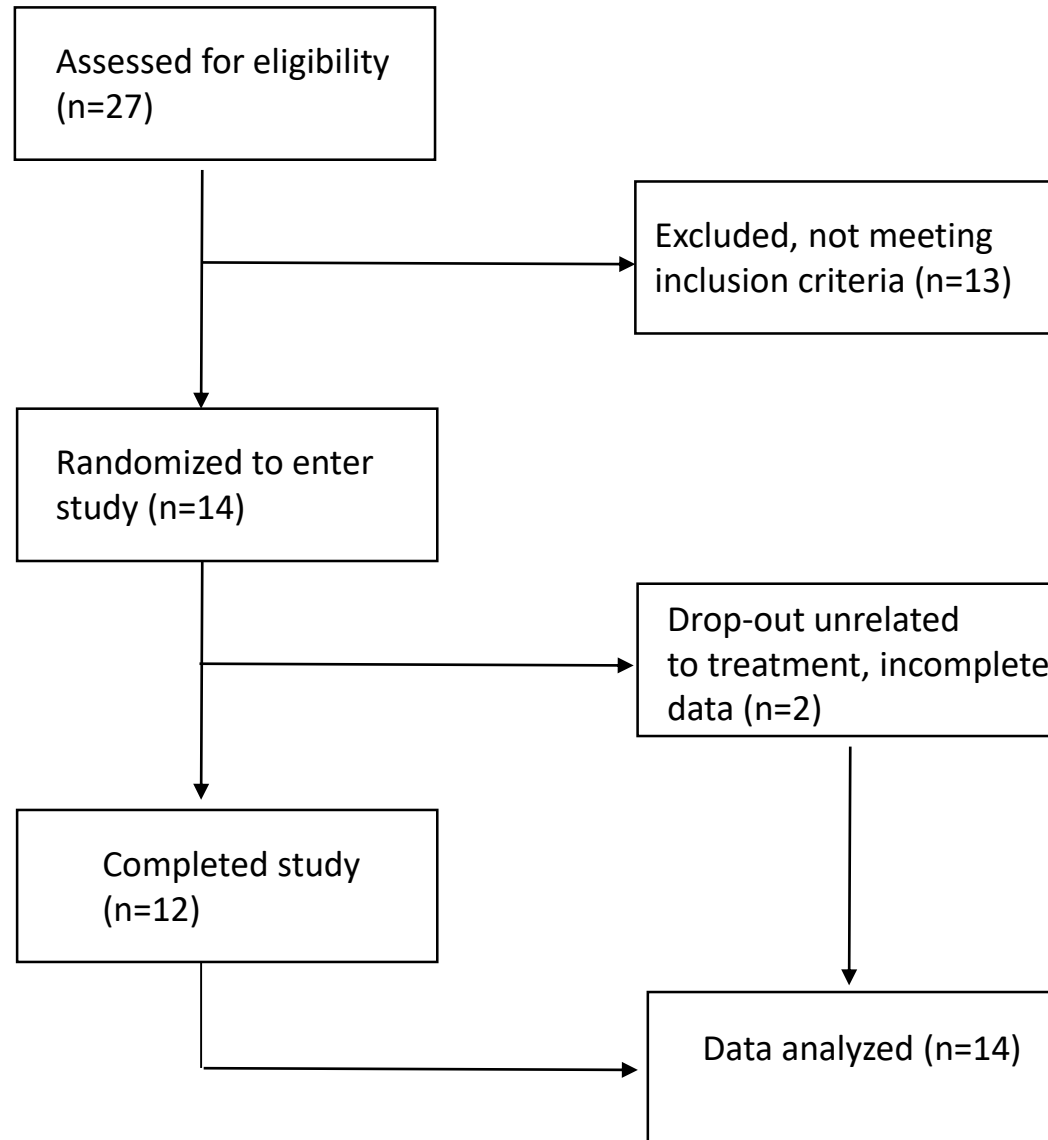

Supplement: Supplementary file 1 — Consort flow chart [file 41386_2024_2042_MOESM1_ESM.pdf]
